# Virucidal Nanofiber Textiles Based on Photosensitized Production of Singlet Oxygen

**DOI:** 10.1371/journal.pone.0049226

**Published:** 2012-11-06

**Authors:** Yveta Lhotáková, Lukáš Plíštil, Alena Morávková, Pavel Kubát, Kamil Lang, Jitka Forstová, Jiří Mosinger

**Affiliations:** 1 Faculty of Sciences, Charles University in Prague, Prague, Czech Republic; 2 Elmarco s.r.o., Liberec, Czech Republic; 3 J.Heyrovský Institute of Physical Chemistry, v.v.i, Academy of Sciences of the Czech Republic, Prague, Czech Republic; 4 Institute of Inorganic Chemistry, v.v.i, Academy of Sciences of the Czech Republic, Řež, Czech Republic; Glasgow University, United Kingdom

## Abstract

Novel biomaterials based on hydrophilic polycaprolactone and polyurethane (Tecophilic®) nanofibers with an encapsulated 5,10,5,20-tetraphenylporphyrin photosensitizer were prepared by electrospinning. The doped nanofiber textiles efficiently photo-generate O_2_(^1^Δ_g_), which oxidize external chemical and biological substrates/targets. Strong photo-virucidal effects toward non-enveloped polyomaviruses and enveloped baculoviruses were observed on the surface of these textiles. The photo-virucidal effect was confirmed by a decrease in virus infectivity. In contrast, no virucidal effect was detected in the absence of light and/or the encapsulated photosensitizer.

## Introduction

The continuing world-wide increase in drug resistance among many classes of pathogenic microbes has created a need for new antibiotic, antiviral and anti-parasitic therapies. Photodynamic therapy is an effective tool for the photoinactivation of bacteria, viruses, fungi and parasites [1], [2], [3] as well as for cancer treatment [4]. The photodynamic effect is due to the oxidative damage caused to biological materials by reactive forms of oxygen, predominantly singlet oxygen, O_2_(^1^Δ_g_), that are generated by photosensitized reactions [5], [6], [7].

Polymeric nanofiber materials, which are commonly prepared from polymer solutions via electrospinning, consist of fibers with diameters in the range of a few nanometers to a few microns [8], [9], [10]. This technique has received substantial attention, especially in the biomedical field, as the high surface area and porous structure of electrospun fibers mean that they can be used as scaffolds for tissue engineering [11]. Electrospun nanofibers can be loaded with different molecules and/or nanoparticles, making them useful tools for a variety of applications, such as the controlled release of drugs and other biologically active species [12], [13], and as antimicrobial agents [14], [15]. Recent studies have described the photobactericidal properties of polyurethane, polystyrene and polycaprolactone nanofiber materials loaded with porphyrinoid photosensitizers [16], [17], [18]. These nanofibers generate O_2_(^1^Δ_g_) and are promising materials for use in the preparation of self-disinfecting wound dressings or filters for water treatment. In contrast to standard anti-bacterial agents, for which continuous release from matrices can lead to diminishing effectiveness over time, these nanofiber materials use atmospheric oxygen and are therefore effective for longer time periods.

In this study, we selected two medical-grade nanofiber materials, polyurethane Tecophilic® and polycaprolactone (PCL), and loaded them with the photosensitizer 5,10,15,20-tetraphenylporphyrin (TPP), which generates O_2_(^1^Δ_g_) with a high quantum yield (Φ_Δ_ = 0.62) upon irradiation [19]. These materials degrade into nontoxic products under physiological conditions, and they are capable of absorbing water, which is essential for optimal wound healing [20]. The previously reported strong photobactericidal effect of O_2_(^1^Δ_g_)-producing nanofiber materials [16], [17] led us to test a similar approach for the photoinactivation of viruses. We used polyomaviruses as models for non-enveloped viruses and baculoviruses as models for enveloped viruses.

The capsid proteins of non-enveloped viruses and the envelope glycoproteins encoded by enveloped viruses enable the viruses to cross plasma membranes into cells and deliver their genetic material to the cell nucleus (or other cellular compartments), resulting in viral gene expression. These proteins are responsible for cell surface receptor recognition and for subsequent interactions with cellular structures, leading to the disassembly of virus particles and the release of genetic information. Therefore, oxidative damage to virion surface proteins via photooxidation of readily oxidizable amino acids (Trp, His, Met and Cys) by O_2_(^1^Δ_g_) may be an effective way to prevent infection [21], [22].

Polyomaviruses, small tumorogenic non-enveloped DNA viruses, have a wide range of hosts, including humans. Two human polyomaviruses, JCV and BKV, which were discovered in 1971, cause progressive multifocal leukoencephalopathy and nephropathy, respectively, in immunosuppressed patients [23], [24]. Since 2007, six new human polyomaviruses (the KI and WU polyomaviruses, Merkel cell polyomavirus, Trichodysplasia spinulosa virus, polyomavirus 6 and polyomavirus 7) have been identified [25], [26], [27]. Merkel cell polyomavirus (MCV or MCPyV), which was described in 2008, is suspected to cause the majority of the cases of Merkel cell carcinoma, a rare but aggressive form of human skin cancer. Baculoviruses, which are large enveloped DNA viruses, are insect pathogens that have been widely used to produce recombinant proteins in cultured insect cells. Baculovirus envelope proteins are also able to mediate entry into human and other mammalian cells and, thus, facilitate the expression of recombinant genes under the transcriptional control of a mammalian promoter. The *Autographa californica* multiple nuclear polyhedrosis virus (AcMNPV), which was used in our experiments, enters cells via a low pH-dependent endocytic pathway [28]. During endocytosis, the major envelope glycoprotein GP64 mediates low pH-triggered membrane fusion, thus releasing nucleocapsids to allow trafficking into the cell nucleus, where the expression of baculoviral genes takes place [29].

## Results

### Morphology and optical properties of the nanofiber materials

**Figure 1:**
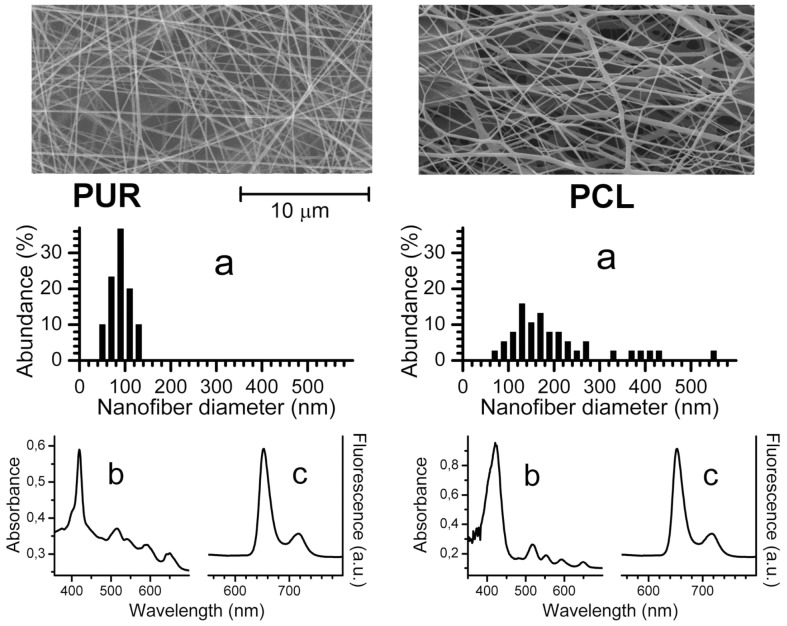
Characterization of the nanofiber materials. Properties of Tecophilic® (first column) and PCL (second column) nanofiber textiles: SEM images with the diameter statistics (a); UV/VIS absorption (b) and fluorescence (c) spectra.

The structure of the nanofiber materials was visualized by scanning electron microscopy (SEM) (Fig. 1). The area weight of the resulting nanofiber textiles was 2 g/m^2^. The average nanofiber diameter (calculated as shown in Fig. 1A) was 89±22 nm for Tecophilic® and 204±106 nm for PCL. The nanofiber textile samples had thicknesses of 93 µm (Tecophilic®) and 320 µm (PCL).

To confirm the encapsulation of TPP in polymer nanofibers, UV/VIS and fluorescence spectra were recorded for the doped nanofiber textiles. The UV/VIS spectra of the Tecophilic® and PCL nanofiber textiles showed Soret bands at 419 nm and 421 nm, respectively, as well as the characteristic Q absorption bands of TPP in the red region (Fig. 1). These spectra are similar to those recorded in nonpolar solvents. Confirming the absorption spectra results, the steady-state fluorescence emission bands are similar when compared with the measurements made in nonpolar solvents. The band maxima are observed at 652 nm and 715 nm for TPP in the Tecophilic® and PCL nanofiber textiles (Fig. 1). The UV/VIS and fluorescence spectra indicate that encapsulated TPP is predominantly present in its monomeric form.

### Photosensitized generation of O_2_(^1^Δ_g_)

**Figure 2:**
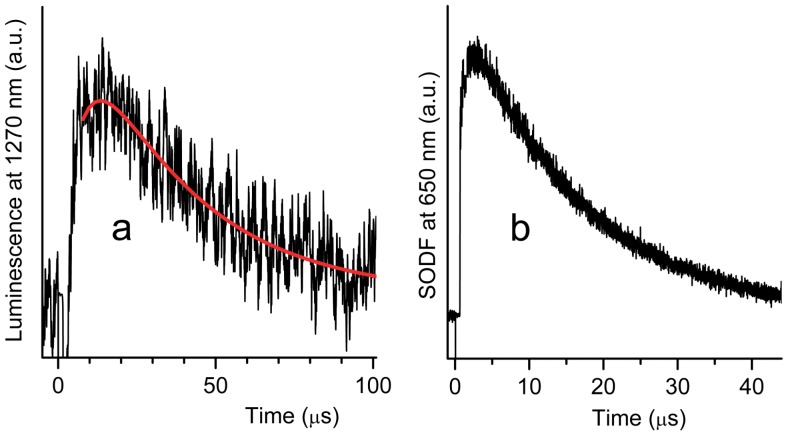
Photogeneration of O_2_(^1^Δ_g_) by the nanofiber textile doped with TPP. Phosphorescence of O_2_(^1^Δ_g_) after excitation of TPP in the Tecophilic® nanofiber textile with a blue light (425 nm, pulse length = 28 ns) in an air atmosphere (a) and corresponding SODF (b). The red curve represents the fitting line determined by the least-squares method, calculated according to Eq. 1.

To confirm the photosensitized generation of O_2_(^1^Δ_g_) in an air atmosphere, the nanofiber textiles were irradiated with a pulse dye laser (λ_exc_ = 425 nm, pulse width 28 ns), and the time-resolved phosphorescence of O_2_(^1^Δ_g_) was detected at 1270 nm (Fig. 2). It should be noted that rise times shorter than 1 µs cannot be measured accurately because of interference from strong TPP fluorescence. The concentration of O_2_(^1^Δ_g_) that is proportional to the phosphorescence intensity follows equation 1 [16]:
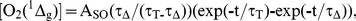
(1)where *A*_SO_ is a parameter proportional to the quantum yield of O_2_(^1^Δ_g_), and τ_T_ and τ_Δ_ are the lifetimes of the TPP triplet states and of O_2_(^1^Δ_g_), respectively.

The fitting process yielded values of τ_T_ = 18±2 µs and τ_Δ_ = 15±3 µs in open air (τ_T_ = 2.9±0.3 µs, and τ_Δ_ = 15±3 µs in a pure oxygen atmosphere) for the Tecophilic® nanofiber material. These values are similar to previously published values for Larithane® polyurethane (τ_T_ = 17 µs, τ_Δ_∼11–21 µs) [16], [18], [30] and polystyrene (τ_T_ = 22 µs, τ_Δ_ = 13 µs) [18]. The TPP triplets in the PCL nanofiber material (τ_T_∼90 µs in open air) were quenched less effectively by oxygen. Analysis of the very weak O_2_(^1^Δ_g_) phosphorescence observed using eq. 1 yielded a value of τ_Δ_ = 10±4 µs.

To visualize O_2_(^1^Δ_g_) generation inside the nanofibers, we measured the singlet oxygen-mediated delayed fluorescence (SODF) that occurred due to the reaction of O_2_(^1^Δ_g_) with TPP triplets inside the polymeric nanofibers (Fig. 2B) [30]. The advantage of this technique compared to direct detection of O_2_(^1^Δ_g_) via phosphorescence is its higher signal-to-noise ratio; however, the kinetics of SODF are complicated and do not allow estimation of lifetimes (τ_T_ and τ_Δ_) through a simple fitting process.

**Figure 3:**
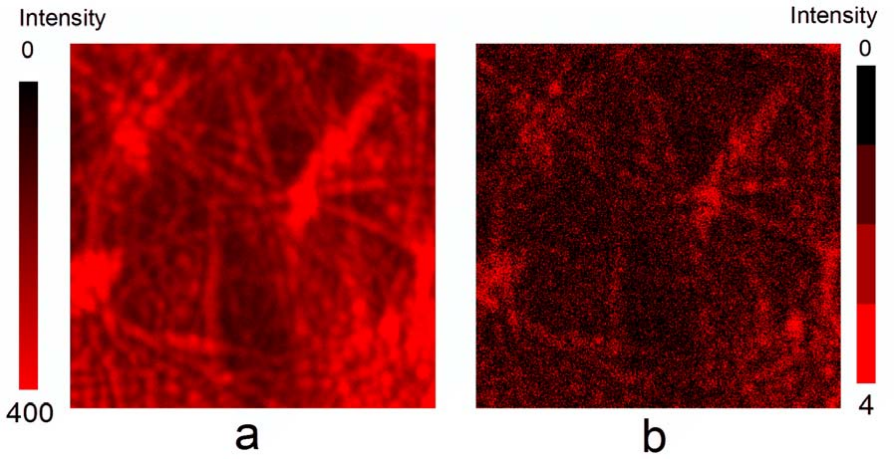
Distribution of TPP molecules in the nanofibers. Confocal fluorescence microscopy: fluorescence intensity images (20×20 µm) of TPP in the Tecophilic® nanofiber textile based on the data collected 10–60 ns after excitation (prompt fluorescence) (a) and 300–2000 ns after excitation (SODF) (b).

Fluorescence lifetime imaging microscopy made it possible to distinguish between the immediate fluorescence that arises from TPP when it is directly excited and the light from SODF, which is dependent on the concentrations of O_2_(^1^Δ_g_) and TPP triplets (Figure 3b) [31]. While the immediate fluorescence intensity image (Figure 3A) shows the distribution of TPP molecules inside nanofibers, the SODF intensity image reveals domains with different concentrations of O_2_(^1^Δ_g_) (Fig. 3B). It should be noted that the diffraction-limited spatial resolution of both images is approximately 200 nm.

The method of O_2_(^1^Δ_g_) imaging using SODF does not monitor O_2_(^1^Δ_g_) outside of the nanofibers. It should be noted that the average diameters of the nanofibers (ca 90 nm for Tecophilic and ca 200 nm for PCL) are sufficiently small for O_2_(^1^Δ_g_) to effectively diffuse outside of the nanofibers and directly interact with viruses. The average diffusion length of O_2_(^1^Δ_g_) depends on the diffusion coefficient in the polymer; a typical value is several tens to hundredths of nm for τ_Δ_ within a range of 10 to 25 µs [31]. Although τ_Δ_ in the surrounding aqueous media falls to 3.1 µs [32], the diffusion length remains unchanged or increases because the diffusion coefficient of oxygen in water is one or two orders of magnitude higher than that in a polymer.

### Photooxidation of 9,10-anthracenediyl-bis(methylene)dimalonic acid (AMA) on the surface of the nanofiber textiles doped with TPP

**Figure 4:**
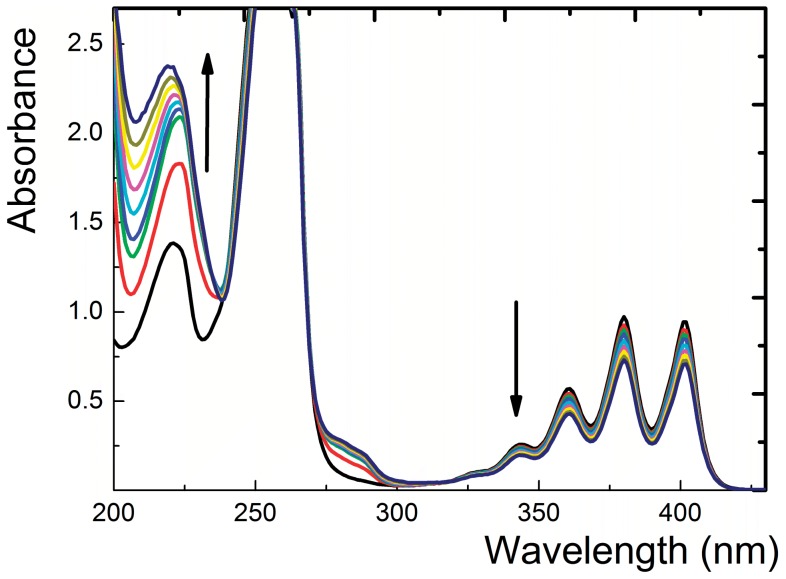
Photooxidation ability of the TPP-doped nanofiber textile. Photodegradation of AMA during 10 min of irradiation of 3 ml of 10^−4^ mol l^−1^ AMA containing a piece of the nanofiber textile doped with TPP (1 cm^2^). The arrows indicate the course of photooxidation. Irradiation was performed using white light from a stabilized 300 W Xe lamp with an optical cut-off filter (λ≥400 nm) at 22°C in air-saturated 0.02 mol l^−1^ phosphate buffer, pH = 7.0.

The results from both luminescence spectroscopy and microscopy described above presented clear evidence of O_2_(^1^Δ_g_) photogeneration inside the polymeric nanofibers. We next asked whether O_2_(^1^Δ_g_) could diffuse from the nanofibers to the textile surface and oxidize a substrate. As a suitable substrate, we selected AMA, a known water-soluble singlet oxygen trap [33]. Continuous visible light irradiation (see Materials and Methods) of a piece of the nanofiber textile immersed in a detection solution of AMA in air-saturated water resulted in significant spectral changes, indicating photooxidation of AMA to corresponding endoperoxides (Fig. 4). No spectral changes were observed in the absence of light or oxygen (the detection solution was bubbled with N_2_). Furthermore, irradiation of the nanofiber textile without TPP photosensitizer did not induce any AMA photoxidation.

### Photovirucidal effect of the nanofiber textiles doped with TPP

We next asked whether the O_2_(^1^Δ_g_) released from the surface of the TPP-doped hydrophilic nanofiber textiles could inactivate viruses. Therefore, we examined the effect of O_2_(^1^Δ_g_) released from the textiles on viruses falling into two different categories: polyomaviruses, the genomes of which are protected by a proteinaceous coat composed of viral capsid proteins (non-enveloped viruses), and baculoviruses, as representatives of enveloped viruses that possess an additional protective envelop composed of a lipid bilayer derived from cellular membranes with incorporated viral transmembrane glycoproteins.

**Figure 5:**
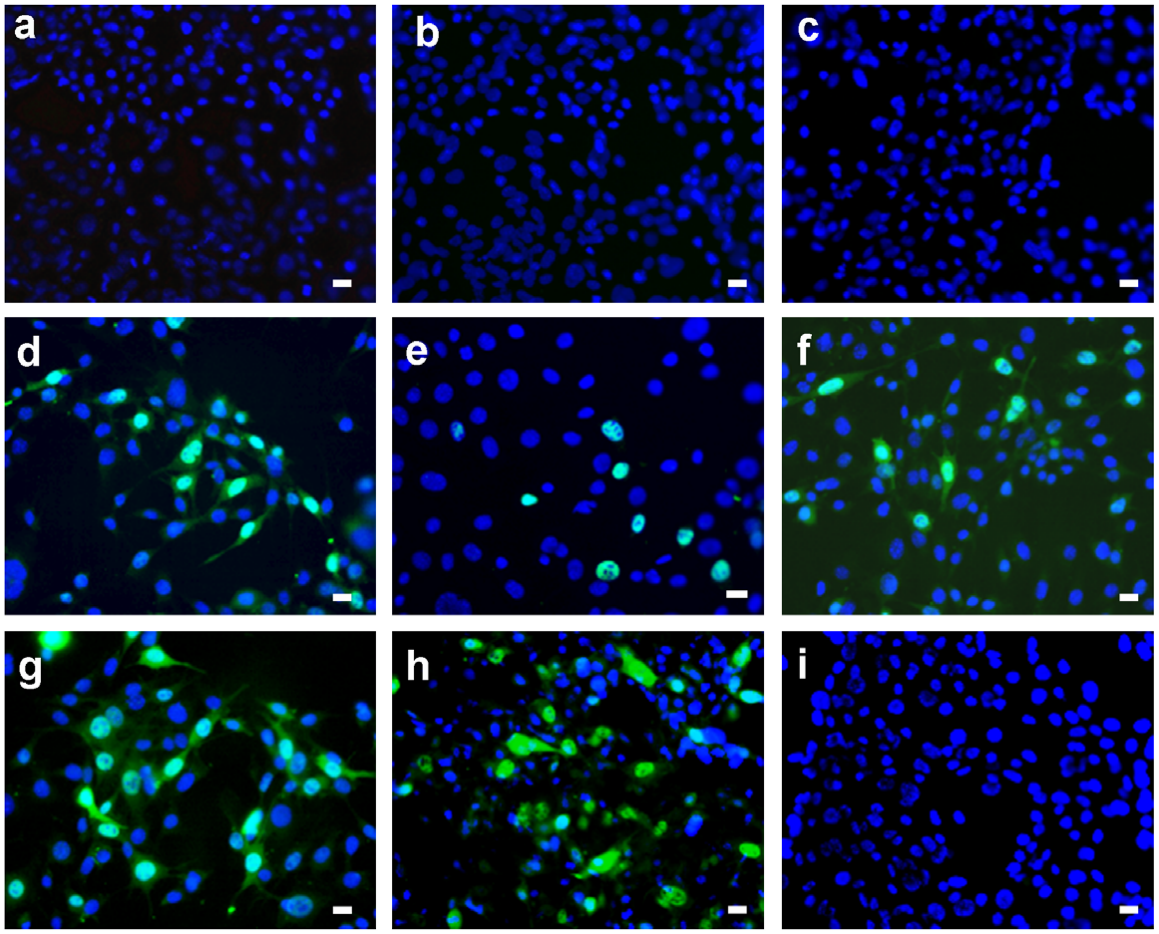
Inactivation of the mouse polyomavirus on the surface of TPP-doped Tecophilic® nanofiber textile. Cells infected with polyomavirus eluate from the surface of the nanofiber textile after 30 minutes of irradiation (a, b, c) or without irradiation (d). Cells infected with control polyomavirus eluate from the surface of the textile without TPP after 30 minutes of irradiation (e) or without irradiation (f). Cells infected with the same amount of the virus in the absence of the textile after 30 minutes of irradiation (g) or without irradiation (h). Non-infected cells (i). Detection of the LT antigen (green) in the nuclei of infected cells. To visualize cell nuclei, DNA was stained with DAPI (blue). Representative images are shown with the bar of 20 µm at the right corner.

To test for potential photo-virucidal activity, the virus inoculum was applied to the surface of small square (1×1 cm) pieces of the nanofiber textiles doped with 1% TPP in a minimal volume to ensure close contact of the virus with the textile. As O_2_(^1^Δ_g_) is produced by the TPP photosensitizer only upon exposure to light, the nanofiber textiles soaked with the virus were irradiated with white light for 30 minutes. Control sets of the textiles soaked with the virus were kept in the dark. Another control set of the textiles without TPP were irradiated under the same experimental conditions. To determine the level of virus inactivation by O_2_(^1^Δ_g_), the virus was retrieved from the surface of the textiles and used to infect 3T6 fibroblasts (for the mouse polyomavirus) and the Sf9 insect cell line (derived from ovary cells of *Spodopterta frugiperda*). These cells were used to measure virus inactivation (see Materials and Methods). Figure 5 shows the effects of O_2_(^1^Δ_g_) produced by the Tecophilic® textiles doped with 1% TPP on the non-enveloped mouse polyomavirus. Infection with the virus was followed by the indirect immunofluorescence analysis of cells using an antibody directed against the large T (LT) antigen, which accumulates in the nuclei of infected cells. While the virus extracted from the textiles with 1% TPP kept in the dark retained the same infectivity (Fig. 5D) as the virus that was not in contact with the doped nanofiber textiles (Fig. 5g), the virus exposed to photogenerated O_2_(^1^Δ_g_) was noninfectious (Fig. 5A, 5B, and 5C). To ensure that the loss of infectivity was not caused merely by virus irradiation and/or slightly increased temperature, the same experiments were performed with the textiles without TPP. Comparable amounts of infected cells were observed when using the virus extracted from the control textiles after a 30-minute irradiation (Fig. 5E) and from the controls kept in the dark (Fig. 5F).

**Figure 6:**
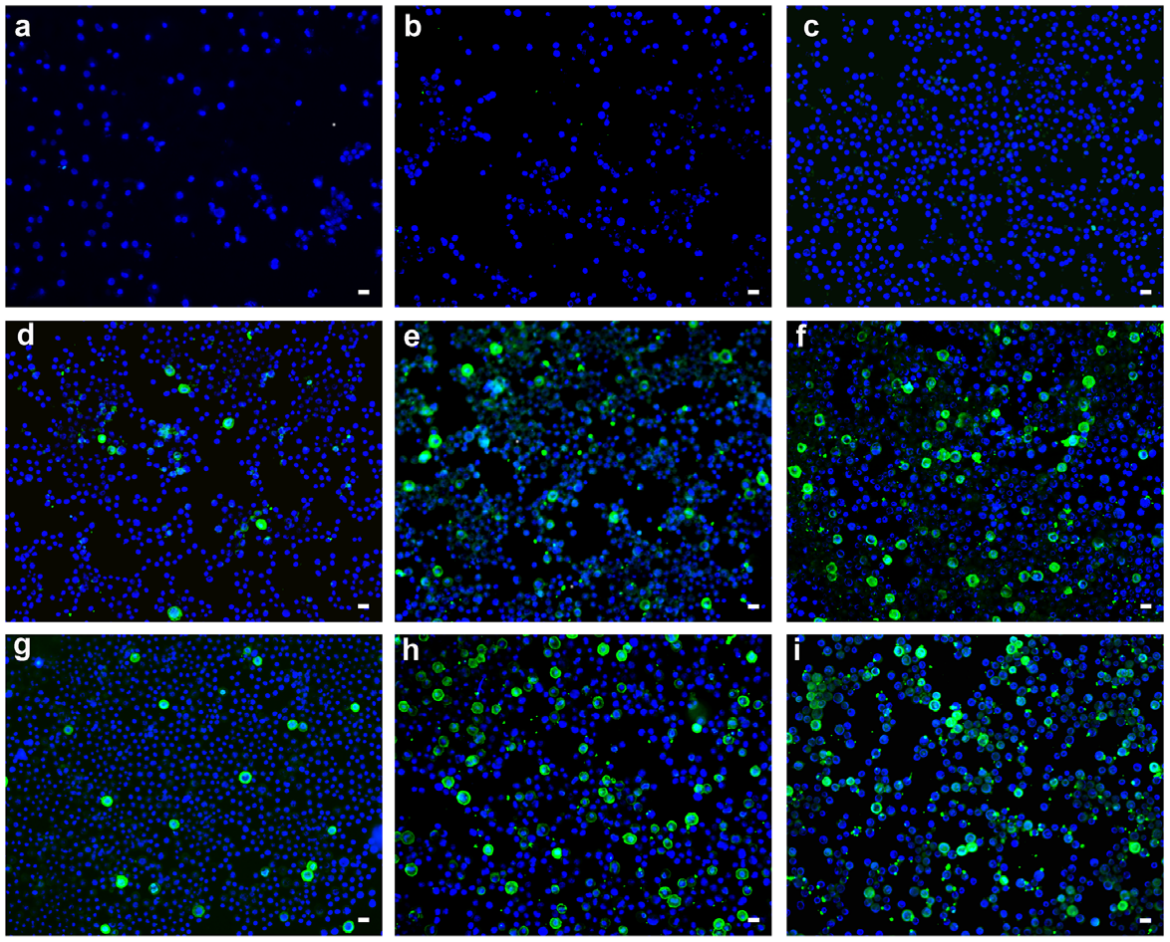
Inactivation of the recombinant baculovirus on the surface of TPP-doped Tecophilic® nanofiber textile. Cells infected with recombinant baculovirus eluate from the surface of the nanofiber textile after 30 minutes of irradiation (a, b, c) or with no irradiation (d, e, f). Cells infected with the baculovirus control eluate from the textile without TPP after 30 minutes of irradiation (g, h, i). MPyV VP1 protein (green) produced from the recombinant baculovirus and DAPI-stained cell nuclei (blue). Different volumes of the viral inoculum (10 µl (a, d, g), 50 µl (b, e, h) or 70 µl (c, f, i)) were applied to the textile. Representative images are shown with the bar of 20 µm at the right corner.

Similar results were obtained with the enveloped baculovirus. Figure 6 shows Sf9 insect cells infected with the recombinant baculovirus extracted from the surface of the Tecophilic® textiles. Thirty-six hours after infection with the virus, the cells were subjected to immunofluorescence detection of the VP1 protein (stained green) produced from the gene inserted into the recombinant baculovirus genome. Thus, O_2_(^1^Δ_g_) released from the surface of textiles upon irradiation inactivates both the non-enveloped and enveloped viruses efficiently.

Analogous results were obtained using PCL textiles for both types of viruses, indicating that both types of polymer nanofibers are sufficiently hydrophilic, are nontoxic to viruses in the absence of light and generate virucidal O_2_(^1^Δ_g_).

**Figure 7:**
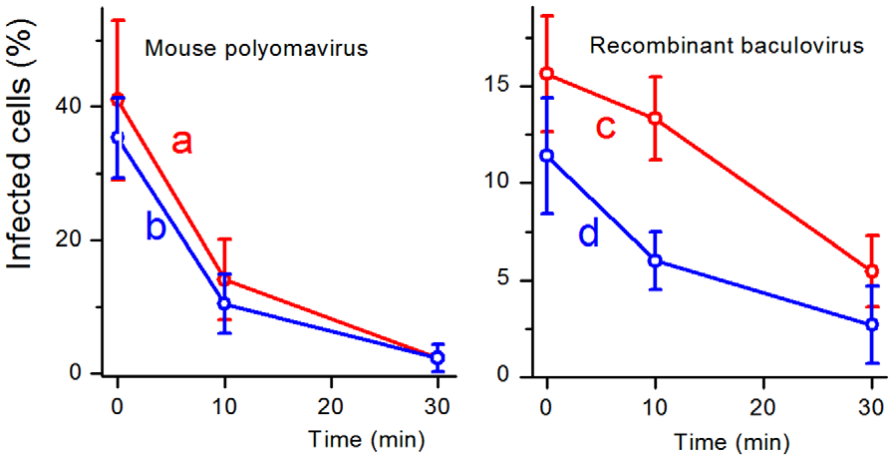
Comparison of virus inactivation on the surface of TPP-doped Tecophilic® and PCL nanofiber textiles. Comparison of inactivation of the mouse polyomavirus (a, b) and the recombinant baculovirus (c, d) by Tecophilic® (a, c) and PCL (b, d) nanofiber textiles doped with 1% TPP. Graphs show percentage of infected cells using virus eluates from the surface of the nanofiber textiles after 0, 10, and 30 minute incubations on the nanofiber textiles exposed to irradiation for indicated times. The values are counted from 5 representative fields containing approximately 130 cells.

Figure 7 compares inhibition of the infectivity of the viruses on the Tecophilic® and PCL textiles doped with 1% TPP after 10 and 30 minutes of irradiation. The inhibition effects were similar and in both cases the baculovirus was more resistant. These results do not fully correlate with photophysical measurements (see above), where the Tecophilic® nanofibers produced O_2_(^1^Δ_g_) in higher yield than PCL ones). The reason could be in the short lifetime of O_2_(^1^Δ_g_) and from this following a limited diffusion length, the factors that are dependent on a surrounding medium. It should be mentioned that the textiles were also tested after a year of storage at room temperature in the dark with the same results. It indicates the long-term photovirucidal efficiency of the both textiles.

**Figure 8:**
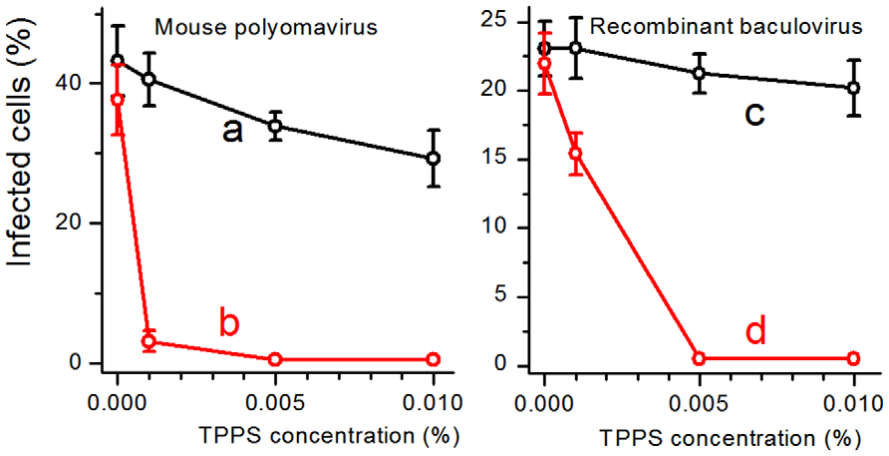
Inactivation of the mouse polyomavirus and the recombinant baculovirus in aqueous solutions of TPPS. Percentages of infected cells by the mouse polyomavirus (a,b) or the recombinant baculovirus (c,d) previously incubated for 30 minutes in solutions of indicated concentrations of TPPS in the dark (a,c) and after irradiation (b,d). The values are counted from 5 representative fields containing approximately 130 cells.

Alternatively, an inhibition effect was found in aqueous solutions of sulfonated analogue TPPS that have the similar quantum yield of O_2_(^1^Δ_g_) as TPP [19] (Fig. 8). The concentration of TPPS above 0.005% entirely inhibited both viruses. At 0.001% TPPS, the infectivity of the mouse polyomavirus was one order of magnitude lower, while the baculovirus was more resistant as its infectivity decreased to approximately 65%.

## Discussion

Singlet oxygen generated in close proximity to living eukaryotic or bacterial cells has been shown to have strong cytotoxic effects [34]. It is well established that the main targets of O_2_(^1^Δ_g_) are cytoplasmic membrane proteins. Integrated proteins that cross the lipid bilayer (with major portions exposed on the cell surface) and peripheral proteins associated with the cell surface have important, often indispensable physiological functions (for instance, acting as protein receptors, pumps, channels or enzymes), and damaging these proteins quickly leads to cell death. Exposure of proteins to O_2_(^1^Δ_g_) can result in oxidation of side-chains, formation of cross-linked/aggregated species, protein unfolding or conformational changes. Aromatic amino acids (tryptophan, tyrosine and histidine) and sulphur-containing amino acids (methionine, cysteine and cystine) are direct targets of O_2_(^1^Δ_g_) [35]. Other O_2_(^1^Δ_g_) targets include unsaturated lipids in the cytoplasmic membrane, which can be oxidized to form lipid hydroperoxides. Oxidation of cholesterol by O_2_(^1^Δ_g_) results in the formation of a number of readily distinguishable oxidation products, especially hydroperoxides [36].

Enveloped viruses possess a lipid bilayer envelope derived from cellular membranes and embedded with viral proteins. These viral surface proteins are often glycosylated and play a crucial role in cell receptor recognition and viral entry into host cells. Therefore, enveloped viruses might be affected by O_2_(^1^Δ_g_) in a manner similar to bacterial and animal cells. Indeed, we showed that the baculoviruses, as representative enveloped viruses, were efficiently inactivated when applied to the surface of the nanofiber textiles doped with 1% TPP and exposed to visible light for 30 minutes. Similar effects may also be expected for other enveloped viruses. The influenza virus envelope contains two surface glycoproteins: hemagglutinin, which is responsible for viral entry into the host cell and its release from endosomes, and neuraminidase, an enzyme that is essential for virus propagation due to its ability to cleave the sialylated virus receptor, thus releasing viral progeny. Zanamivir, a neuraminidase inhibitor, is one of drugs used to treat patients infected with the influenza virus. Wen-Hsien Wen et al. [37] showed that tetrameric zanamivir conjugates based on a porphyrin core structure, despite being less potent in inhibiting neuraminidase, are significantly more potent in inactivating influenza viruses. The authors attribute this effect to the high local concentration of the photosensitizer porphyrin, which generates O_2_(^1^Δ_g_) in a close proximity to the virus surface.

The nucleic acids in non-enveloped viruses are enclosed in protective, protein-only capsids. Capsids in non-enveloped viruses have simple symmetric structures and are formed from many identical subunits composed of one or several proteins. The protein-protein interactions among these subunits maintain a tightly packed, stable capsid structure that is able to survive exposure to extreme pH levels, harsh environmental conditions, proteolytic enzymes, or even strong detergents in some cases.

**Figure 9:**
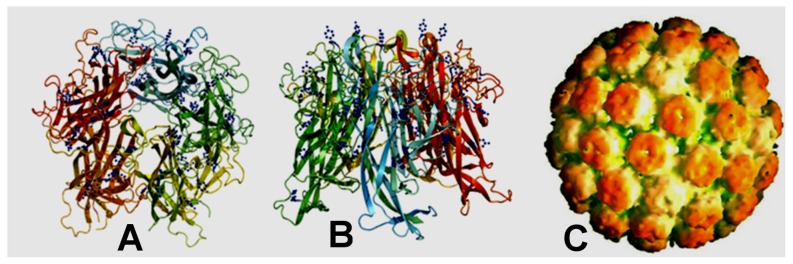
Structure of the polyomavirus capsid. Structure of the polyomavirus capsid (C) formed by 72 pentamers of the major structural protein VP1. A, B: Two orientations of VP1 pentamers with tyrosines (blue) exposed on the surface of VP1 loops. Program: PyMOL Version 0.99rc6, DeLano Scientific LLC, 2006, http://www.ncbi.nlm.nih.gov/pubmed/.

The crucial requirement for O_2_(^1^Δ_g_)-mediated protein damage to occur efficiently is localization of amino acid residues sensitive to O_2_(^1^Δ_g_) on the surface of compact capsid structures. The crystal structures of MPyV and Simian virus 40 (SV40) have been determined. The capsid shell of the polyomavirus used in this study is composed of 72 pentamers of the major structural protein VP1 (Fig. 9). Two other minor structural proteins, VP2 and VP3, are not exposed on surface of the capsid. VP1 from both polyomaviruses contains a β-sandwich core with several outfacing loops [38], [39]. These interactive loops are exposed on the surface of VP1 pentamers and polyomavirus capsids. Computer analysis revealed the presence of several tyrosine and tryptophan residues as well as one histidine and one methionine residue in the surface loops. Many other sensitive amino acid residues occurring in the VP1 β-sandwich core might be less accessible.

The level of accessibility of the amino acid residues that are sensitive to O_2_(^1^Δ_g_) can differ among capsid proteins of non-enveloped viruses, and it will be necessary to test the efficiency of their inactivation individually. Recently, efficient inactivation of the non-enveloped bacteriophage MS-2 by visible light was reported based on using a cationic fullerene derivative with amine functionality as a photosensitizer to produce O_2_(^1^Δ_g_) [40]. Based on the computer analysis of capsid subunits from viruses with known tertiary structures, we predict that human papillomaviruses or poliovirus can be efficiently inactivated by O_2_(^1^Δ_g_) produced by the photosensitizer used in this study. Thus, the photosensitizers immobilized on the nanofibers can be highly useful for the development of novel approaches for inactivating both enveloped and non-enveloped viruses.

## Conclusions

This study, addressing the photophysical, photochemical and photovirucidal properties of polymer nanofibers based on the Tecophilic® thermoplastic polyurethane and polycaprolactone with an encapsulated 5,10,5,20-tetraphenylporphyrin photosensitizer, reveals that these textiles are efficient sources of short-lived virucidal O_2_(^1^Δ_g_). The photoproduction and lifetime of O_2_(^1^Δ_g_) in these materials are sufficient to exert strong photovirucidal effects on non-enveloped polyomaviruses and enveloped baculoviruses on the surface of the nanofiber textiles. These new nanomaterials could be considered for use in a number of medical applications and for the development of O_2_(^1^Δ_g_) inactivation tests for enveloped and non-enveloped viruses.

## Materials and Methods

### Chemicals

5,10,15,20-tetraphenylporphyrin (TPP), 5,10,15,20-tetrakis(4-sulfonatophenyl)porphyrin (TPPS), 9,10-anthracenediyl-bis(methylene)dimalonic acid (AMA) and tetraethylammonium bromide (TEAB) were purchased from Aldrich (USA). Formic acid, acetic acid, N,N-dimethylacetamide (DMAc) and toluene were purchased from Penta (Czech Republic). Polyurethane Tecophilic® HP-60D-60, a thermoplastic elastamer consisting of segmented block copolymers and synthesized from 4,4′-diisocyanato dicyclohexylmethane, 1,4-butanediol and polyethylene glycol, was purchased from Lubrizol Advanced Materials (USA). Polycaprolactone (MW 70,000) was purchased from Scientific Polymer Products, Inc. (USA). For DNA staining, 4,6′-diamidino-2-phenylindole, dihydrochloride (DAPI) was purchased from Sigma (USA).

### Preparation of the Tecophilic® and PCL nanofiber materials

Nanofiber materials were produced using the Nanospider™ electrospinning technology [41]. The solution used to prepare the Tecophilic® nanofiber material (8% in DMAc∶toluene, 2∶1 w/w) contains 1 wt % TPP, 0.01 wt % TEAB and 98.99 wt % Tecophilic®. The solution used to prepare the PCL nanofiber material (15% in formic∶acetic acid, 1∶3 w/w contains 1 wt % TPP and 99 wt % PCL.

### Absorption and fluorescence spectroscopy

The UV/VIS absorption and fluorescence spectra were recorded on Perkin Elmer Lambda 35 and Fluorolog 3 (Horiba Jobin Yvon) spectrophotometers, respectively. The samples were excited at the band maximum of TPP (413 nm).

### O_2_(^1^Δ_g_) phosphorescence

The nanofiber materials were excited using a Lambda Physik FL 3002 dye laser (425 nm, pulse width 28 ns). Time-resolved near-infrared phosphorescence of O_2_(^1^Δ_g_) at 1270 nm was observed at a right angle to the excitation pulse using a homemade detector unit (interference filter, Ge diode Judson J16-8SP-R05M-HS). The incident energy used is the region where the intensity of a phosphorescence signal is directly proportional to the incident energy (less than 1 mJ). A singlet oxygen signal was corrected using detector responses in vacuum to eliminate fluorescence and scattered light.

### Singlet oxygen-mediated delayed fluorescence

SODF was recorded using an LKS 20 kinetic spectrometer (Applied Photophysics, UK). The samples were excited with the same laser that was used for phosphorescence measurements [30], [31]. The fluorescence time profiles were recorded at 460 nm using an R928 photomultiplier (Hamamatsu). SODF was calculated as the difference between TPP fluorescence in an air (oxygen) atmosphere and in a vacuum.

### Confocal fluorescence and fluorescence lifetime imaging microscopy

These measurements were carried out using a MicroTime 200 inverted epifluorescence confocal microscope (PicoQuant, Germany) [31]. The configuration used in these analyses included a pulsed diode laser (LDH-P-C-405, 405 nm, PicoQuant) providing 80 ps pulses with a repetition rate of 40 MHz, a 505DRLP dichroic mirror, an LP500 long-pass filter (Omega Optical), a water immersion objective (1.2 NA, 60×) (Olympus) and a PDM SPAD detector (MPD, USA).

### Continuous irradiation of the nanofiber materials in the presence of AMA

A piece of the nanofiber material was peeled off of the supporting polypropylene textile, coiled around a quartz plate (10×40×1 mm), and inserted into a thermostatted 10 mm quartz cell (22°C) containing a 10^−4^ M aqueous solution of AMA. The cell was irradiated using a 300 W stabilized Xe lamp with an optical cut-on filter (λ≥400 nm). The changes in UV/VIS absorbance due to the formation of oxidized products were recorded at regular time intervals and compared with the changes observed in a blank solution without irradiation.

### Viruses and cells

*Spodoptera frugiperda* cells (Sf9) were cultured as a monolayer at 27°C in TNF-FH medium containing 10% fetal calf serum (FCS), as described by Hink [42]. The recombinant baculovirus pVL-VP1, carrying the mouse polyomavirus VP1 gene driven by a polyhedrine promoter, was used to infect insect cells [43]. Swiss Albino mouse 3T6 fibroblasts were grown at 37°C in a 10% CO_2_ air humidified incubator using Dulbecco's modified Eagle's medium (DMEM) supplemented with 2 mM glutamine and 10% fetal calf serum (FCS).

Mouse polyomavirus (strain A2) was propagated for 7 days in whole mouse embryo cells (0.05 PFU per cell). Virions were purified according to Türler and Beard [44].

### Antibodies

Two different primary antibodies were used: a mouse monoclonal antibody against the mouse polyomavirus VP1 protein [43] and a rat monoclonal antibody against the mouse polyomavirus large T (LT) antigen [45]. Alexa Fluor 488 (green)-conjugated goat anti-mouse or donkey anti-rat immunoglobulin antibody was used as a secondary antibody.

### Treatment of the mouse polyomavirus on the surface of nanofiber textiles doped with TPP

Mouse polyomavirus in DMEM (100 µl; 1×10^5^ plaque forming units (PFU)) was dropped onto 1.0 cm^2^ of the nanofiber textile (polyurethane Tecophilic® or PCL), placed on Parafilm in a dish cooled by ice and irradiated (as described above) or kept in the dark. The liquid containing the virus was collected from the nanofiber textiles; 2×100 µl of DMEM was added to extract the remaining virus; and the combined virus fractions were transferred to a 24-well dish containing 3T6 fibroblasts grown on coverslips.

### Treatment of the baculoviruses on the surface of nanofiber textiles doped with TPP

Baculoviruses in TMN–FH insect medium (Sigma) (25 or 50 µl; approx. 5×10^4^ PFU) were applied to nanofiber textiles and treated as described above. The liquid containing the virus was then collected from the surface of the nanofiber textiles; 2×100 µl of insect medium was added to extract the remaining virus; and the combined virus fractions were transferred to a 24-well dish containing Sf9 cells grown on coverslips.

### Treatment of the mouse polyomavirus and the recombinant baculovirus in TPPS solutions

Viruses were incubated in 100 µl of appropriate media; (see above) containing different concentrations of water-soluble TPPS (0, 0.001, 0.005, 0.010%) for 30 minutes either in darkness or upon irradiation. The media containing the viruses were then used for infection of the cells growing on coverslips.

### Viral infection of cells

Adsorption of the polyomavirus to the surface of the 3T6 cells was performed by incubating the virus and cells together for 1 hr at 0°C. Then, 1 ml of pre-warmed DMEM containing 10% FCS was added to each well, following which the cells were incubated at 37°C in a 10% CO_2_ air humidified incubator for 20 hours and finally fixed.

Adsorption of the baculovirus to the surface of the Sf9 cells was performed by incubating the virus and cells together for 1 hr at room temperature. The medium was then removed, and 1 ml of pre-warmed TMN–FH insect medium containing 10% FCS was added to each well, following which the cells were incubated at 27°C for 36 hours and subsequently fixed.

### Immunofluorescence

Cells were fixed with 3% paraformaldehyde for 30 minutes, followed by incubation with 0.5% Triton X-100 for 5 minutes at room temperature. Nonspecific antibody binding sites were blocked via a 30-minute incubation in PBS (140 mM NaCl, 2.7 mM KCl, 10 mM Na_2_HPO_4_, 1.8 mM KH_2_PO_4_; pH = 7.3) containing 0.25% gelatin and 0.25% bovine serum albumin. Then, the cells were incubated for 30 minutes with a specific rat monoclonal antibody directed against the large T antigen (for mouse polyomavirus) or a mouse monoclonal antibody against the polyomavirus VP1 protein produced by recombinant baculovirus (for the baculovirus). Unbound antibody was removed by washing with PBS (3×10 minutes), and the cells were then incubated for 30 minutes with a secondary antibody conjugated with Alexa Fluor 488 directed against a rat or mouse immunoglobulin. The cells were finally washed with PBS (3×10 minutes), and cover slips were mounted with glycerol with DAPI. Infected cells were visualized by fluorescence microscopy using Lucia Software (version 5.1.), Laboratory imaging s.r.o., Prague, Czech Republic.
